# Suppress to get along: a lifespan account of social motives for suppression at work

**DOI:** 10.1007/s11031-025-10146-w

**Published:** 2025-10-06

**Authors:** Elissa El Khawli, Anita C. Keller, Susan Reh, Susanne Scheibe

**Affiliations:** 1 Open University of the Netherlands, Heerlen, The Netherlands; 2 University of Groningen, Groningen, The Netherlands; 3 University of Exeter Business School, Exeter, United Kingdom

**Keywords:** Suppression, Incivility, Social motives, Work context, Worker age

## Abstract

Expressive suppression, an emotion regulation strategy that entails hiding one’s feelings, is commonly used to deal with interpersonal stressors at work, despite its negative relationship with affective well-being. To understand the context around suppression at work, we adopted a motivated, situational emotion regulation perspective to investigate the motives and context of suppression use at work. In a daily diary study, we investigated the effects of experienced incivility, in concert with daily communion and status motives, in predicting suppression at work. Drawing on lifespan theories, we also addressed between-person differences in communion and status motives by investigating how age relates to these motives via two theoretically established developmental goal orientations - growth and maintenance. Data were analyzed from 291 participants who participated in a daily diary study with three daily measurements for 15 working days (3,159 daily records). At the within-person level, incivility and communion motives both predicted use of suppression. Status motives did not relate to suppression, nor did either motive interact with experienced incivility to predict suppression. At the between-person level, age was indirectly negatively related to status and communion motives via lower growth orientation. Our findings offer insights into how daily motives influence emotion regulation strategy use, as well as how age and developmental goal orientations relate to these motives at the inter-person level.

## Introduction

Expressive suppression is an emotion regulation strategy that is focused on regulating the expression of the emotion instead of the emotional experience itself (Gross, 2015). This strategy is frequently employed as a response to social work stressors (Diefendorff et al., 2008; Scheibe & Moghimi, 2021; Sliter et al., 2010). Indeed, a person who experiences incivility (i.e., rude or uncivil behavior from others) at work may want to suppress the expression of their negative emotions, for example, to prevent exacerbating a conflict with the instigator (Bolino et al., 2016; Scheibe & Moghimi, 2021). Yet, suppression is a double-edged sword: In the short term, suppressing emotions to avoid conflicts may be beneficial, but in the long run, it could have a negative impact on the regulator’s well-being (English, 2024; Gross, 1998). Empirical and meta-analytic findings show that expressive suppression, especially when used habitually, is associated with unfavorable affective and social well-being outcomes (Brockman et al., 2016; Chervonsky & Hunt, 2017; Gross, 2015). Reflecting suppression’s double-edged consequences, the literature has shifted from considering suppression (and other regulatory strategies) as putatively adaptive or maladaptive towards examining its contextual utility and functionality (Sheppes, 2020). In line with this perspective and specifically focusing on the organizational context, this study’s goal was to better understand (1) the situational and motivational context that prompts people to suppress their emotions at work, specifically the context of incivility and work-related social motives, and (2) for which employees these motives are prevalent.

As suppression is used in social contexts (e.g., Scheibe & Moghimi, 2021), we focus on the role of two fundamental social motives as predictors of suppression use at work, namely communion (the desire to get along) and status motives (the desire to get ahead) (Anderson et al., 2015; Barrick et al., 2002). Although motives are commonly conceptualized as trait-level constructs (e.g., Barrick et al., 2002, 2013), they may also fluctuate on a daily level, depending on work events and circumstances that afford them (e.g., Scott et al., 2014; Sonnentag et al., 2017). Consequently, we conceptualize incivility, daily communion motive, and daily status motive as within-person predictors of suppression use at work. Moreover, we investigate whether said motives modulate the extent to which workers use suppression following an experience of incivility.

Further, we investigate for whom communion and status motives are prevalent (i.e., at the between-person level). A potentially important factor influencing the average prevalence of social motives at work is employees’ age. Lifespan theory suggests that aging is accompanied by a reorganization of motivations and priorities in different developmental contexts, causing a shift from a growth orientation (allocating resources to achieve higher levels of functioning) towards a maintenance orientation (allocating resources to maintain stable levels of functioning in the face of losses and challenges) (Baltes et al., 2007). We argue that in the work context, an untested implication is that these general developmental tendencies may shift the prevalence of communion and status motives in workplace interactions (also see Kanfer & Ackerman, 2004). Accordingly, we examine how age affects social motives that relate to daily suppression use through the broad developmental goal orientations of growth and maintenance. In doing so, we move towards a perspective that examines the broader context of emotion regulation strategy use, as a stepping stone towards understanding the functionality of emotion regulation.

## Within-person level: daily incivility, social motives, and suppression at work

### Daily experiences of incivility and suppression

Workplace incivility is defined as a “low-intensity deviant behavior with ambiguous intent to harm the target, in violation of workplace norms of mutual respect” (Andersson & Pearson, 1999, p. 457). It comprises behaviors such as being rude or disrespectful to others and addressing them unprofessionally (Cortina & Magley, 2003). Despite its low-intensity nature, incivility poses a threat to self-esteem and is significantly associated with detrimental well-being outcomes (Cortina et al., 2001; Gerhardt et al., 2021; Mao et al., 2019).

Previous research has found that experienced incivility elicits the use of surface acting, an emotion regulation strategy that is similar to suppression (e.g., Sliter et al., 2010) in that it also involves the suppression of emotional expressions, in addition to the expression of inauthentic emotions (Grandey, 2003). Whereas surface acting is used specifically to manage one’s emotional displays to match organizational display rules (emotional displays required by the organization; e.g., Grandey, 2003), suppression, as any form of inhibitory behavior, may be linked to any situation in which people feel low in social standing or power (Catterson et al., 2017; Cho & Keltner, 2020). This is because high power affords people more freedom, and therefore encourages them to engage in more approach behaviors, including expressive behaviors. Low power, in contrast, constrains people’s behavior and activates their inhibitory tendencies (Cho & Keltner, 2020; Keltner et al., 2003). Accordingly, incivility may encourage the use of suppression because it makes people feel disrespected, thereby threatening their sociometric standing at work. Additionally, workers may be encouraged to suppress their emotions following an experience of incivility to save face in front of colleagues and maintain a professional façade. We thus propose the following:

#### Hypothesis 1

Daily incivility is positively related with daily use of suppression.

### Daily social motives and suppression

Motives are higher-order goals that direct behavior and cognitions (Barrick et al., 2002, 2013). Two common motives in interpersonal (work) contexts are communion and status (Barrick et al., 2002). Communion refers to the motive of gaining acceptance from others and getting along with them, whereas status refers to getting ahead of others at work in a way that makes others esteem them (Anderson et al., 2015; Barrick et al., 2002). As with other forms of self-regulatory behavior, motives steer people towards regulating their emotions to achieve outcomes that are congruent with these motives (Bonanno & Burton, 2013; Inzlicht et al., 2021; Tamir, 2016). For example, after a colleague mistreats them at work, a person who strives for communion may regulate their emotions by suppressing them, as this may help maintain harmony among colleagues, whereas a person who strives for high status may seek confrontational regulatory strategies to restore their sense of status. Work motives vary both at the trait level (e.g., Barrick et al., 2002, 2013) and also fluctuate at the daily level, depending on the situational circumstances that are salient on that day (e.g., Scott et al., 2014; Sonnentag et al., 2017). Daily motives thus represent the outcome employees want to achieve for that day.

### Communion motive

Suppression may undermine social functioning in close personal relationships because it limits authenticity and emotional sharing (Fischer & Manstead, 2008). Eldesouky and English (2019) find a null relationship between prosocial motives pertaining to close relations and suppression across three studies (for a meta-analysis, see Chervonsky & Hunt, 2017). However, suppression can be functional in the work context as relationships at work are less personal. Suppression may thereby support social harmony and professionalism in this particular context where emotional restraint is more valued (see Ehrhardt & Ragins, 2019; Liu et al., 2010; Semmer et al., 2016). Indeed, displaying negative emotions at work may be contrary to the encouraged positive emotional tone that is common in organizations. Therefore, being too emotionally expressive of negative emotions may be seen as inappropriate (Diefendorff & Richard, 2003). Thus, when people prioritize getting along with others at work (i.e., activated communion motive), they should be more likely to use suppression upon encountering unpleasant aspects of their work. We therefore propose:

#### Hypothesis 2a

Daily communion motive is associated with higher use of suppression.

### Status motive

People who strive for status are thought to attain it by challenging the status quo. Indeed, challenging the status quo may signal competence and agency in the eyes of others, which enhances status perceptions (Anderson et al., 2015). Empirical findings provide support for this position, by showing that engaging in voice behaviors at work (e.g., speaking out, suggesting improvements) leads to higher scores on coworker-rated social status (Weiss & Morrison, 2019). Further, people who strive for status are likely to avoid behaviors that might signal that they have low status, such as behaviors that maintain the status quo (Anderson et al., 2015). Accordingly, striving for status should be related to lower use of expressive suppression, as this strategy is geared towards the maintenance of the status quo (Solak et al., 2021). Therefore, on days with a salient status motive, people should avoid the use of suppression. We therefore propose:

#### Hypothesis 2b

Daily status motive is associated with lower use of suppression.

Daily motives may be most impactful in the presence of social stressors such as workplace incivility. Indeed, incivility signals a threat to one’s communion and relatedness (Semmer et al., 2019). Accordingly, incivility-related threats should be even more prevalent on days when people have a salient communion motive. In such circumstances, people might resort to more suppression to hide their negative emotions and improve their likeability as a coworker. The link between incivility and suppression should therefore be stronger on days when people have a strong communion motive. Additionally, incivility makes people feel disrespected, which signals a threat to their status (Cortina et al., 2001; Leary, 2005). Status threats should lead to overt responses, such as expression of discontent, as these responses may help people stand out and mark their boundaries, and therefore restore their status (Anderson et al., 2015; Fischer & Manstead, 2008). Accordingly, when the status motive is salient, experiencing incivility should lead to the expression, as opposed to the suppression, of workers’ discontent.

#### Hypothesis 3a

Daily communion motives moderate the link between incivility and suppression, such that the link between incivility and suppression is stronger on days when people have stronger communion motives.

#### Hypothesis 3b

Daily status motives moderate the link between incivility and suppression, such that the link between incivility and suppression is weaker on days when people have stronger status motives.

## Between-person level: age, developmental goal orientations, and aggregated levels of social motives at work

### Age and developmental goal orientations

Although social motives can vary from one day to the next, they also vary on the between-person level (Barrick et al., 2013). For example, the prevalence and strength of work-related (social) motives may – at the between-person level – be influenced by people’s career concerns and goal orientations at a given point in their life or career (Kanfer & Ackerman, 2004; Thrasher et al., 2018). Accordingly, to understand for whom suppression-eliciting motives may be prevalent, it is important to understand how the working lifespan brings about changes in overarching goal orientations. Freund and Riediger (2001) argue that during young adulthood, people are motivated to acquire new resources that are needed for their further development. Therefore, resource gains and growth goal orientations that may be beneficial in the future are more salient in young adulthood. In middle age, however, goals related to the conservation of resources become more salient to prevent and counter losses, and to preserve existing resources. Consequently, resource conservation and maintenance goal orientations that may be beneficial in the present are more salient in midlife and older adulthood (also see Ebner et al., 2006).

In the work context, although older workers are still relatively young compared to older adults in general (i.e., a large portion of whom may be retired), they are a subgroup of workers who see their retirement as nearing. Accordingly, these developmental changes regarding growth and maintenance goal orientations should translate into shifts in certain career concerns (Baruch & Bozionelos, 2011). Younger workers may be more future-focused and prefer to grow their work selves by investing in the acquisition of new skills and resources (also see Carstensen et al., 1999). Older workers, in contrast, may be more present-focused, and prefer to maintain what they already have and drawing on their acquired skills and resources (Kochoian et al., 2017). We therefore argue that as workers age, they may be less likely to allocate their resources in the pursuit of growth and career advancement and more likely to allocate their resources in the pursuit of career maintenance (Kanfer & Ackerman, 2004; Scheibe & Kooij, 2024). We propose:

#### Hypothesis 4a

Age is negatively related to growth goal orientations.

#### Hypothesis 4b

Age is positively related to maintenance goal orientations.

### Communion motive

Driven by their salient growth goal orientation, young employees may be primarily concerned with gaining new resources (Freund & Riediger, 2001) to establish themselves and progress within their field or organization. At the same time, younger workers need to navigate a vast amount of uncertainty regarding their career and organizational development. Therefore, they may value integration and social embeddedness in their work environment and organization (i.e., communion motive), as this likely helps them access information and resources that are valuable for career success and advancement (Morrison, 2002). Indeed, empirical evidence shows that having the right social ties within one’s work team can help newcomers perform well (Yuan et al., 2020). Additionally, building one’s relationship with one’s mentor may, in some cases, be related to greater information sharing from the mentor, which can help newcomers adjust to their new organization (Zheng et al., 2021). Therefore, younger workers, driven by their growth goal orientation, may find value in building harmonious social relationships and collaborations at work, and, hence, strive for communion. Indeed, research shows that communal goals are linked to more relationship building and networking behaviors at work which are positively associated with social and organizational learning (Tan et al., 2016). Meta-analytic findings also show that prosocial motivation at work is beneficial for work performance and career success (Liao et al., 2022).

Striving towards communion is more nuanced for older workers with two opposing mechanisms at play. On the one hand, based on socioemotional selectivity theory (SST; Carstensen et al., 1999), older adults prioritize socially meaningful interactions that foster generativity, belongingness, and emotional intimacy as opposed to instrumental social interactions that may have gains in the future. These emotionally meaningful interactions may be more easily found among close others, outside the work context. For example, Antonucci and Akiyama (1987) posit that as people age, they are more likely to rely on very close connections (e.g., family and close friends) and less likely to rely on more peripheral connections, which may include acquaintances, colleagues, and clients. This suggests that older workers may disengage from communion motives at work as their communion and affiliation needs may be met outside the work context. Inceoglu et al. (2012) did not find robust evidence for a positive relationship between age and affiliation motives at work. Similarly, Kooij et al. (2011) found a non-significant meta-analytic relationship between age and general social motives. More importantly, age was negatively correlated with the affiliation motive, or the motive to work with others. Accordingly, older workers, who tend to be present-oriented and not motivated by growth (Baltes et al., 2007; Carstensen et al., 1999), may be less likely to be motivated by communion at work.

On the other hand, maintenance orientation, which is likely more salient among older workers, prompts behaviors that allow people to protect and preserve levels of functioning, especially when faced with a challenge (Baltes et al., 2007). Maintenance may consist of retaining usual productivity levels despite new challenges (e.g., new technology demands), maintaining well-being, or maintaining one’s social network at work. Accordingly, older workers, driven by their maintenance goal orientation, may be motivated to collaborate with colleagues at work to maintain their work network, and to capitalize on their resources and skills to help others (Kooij et al., 2011). Furthermore, older workers, given their limited time perspective, may be more motivated to maintain their legacy and what they have built or achieved in their work environment by passing on their skills to others (Doerwald et al., 2021). They may therefore strive for communion with (younger) colleagues at work in order to achieve these goals. Indeed, Doerwald et al. (2021) found a positive meta-analytic relationship between age and generativity motives, a prosocial motive geared towards coaching and transferring one’s knowledge to others. Similarly, Kooij et al. (2011) found a positive meta-analytic relationship between age and the motive to help others. Accordingly, older workers’ maintenance goal orientation may drive them towards communion striving that is geared towards helping, coaching, and passing their skills on to others. All in all, we posit that the link between age and communion striving at work is not straight-forward, because two countervailing forces are at play: age-related maintenance goal orientations should foster more communion striving with others at work, while age-related disengagements from growth goal orientations should foster less communion striving with others at work. Based on these considerations, we propose:

#### Hypothesis 5

Age is indirectly related to aggregated levels of the communion motive, such that: (a) age is negatively related to the communion motive through its negative association with growth goal orientation, and (b) age is positively related to the communion motive through its positive association with maintenance goal orientation.

### Status motive

Career success, including promotions, often involves competition. Therefore, younger workers, motivated by their growth goal orientation, may be motivated to climb the career ladder, advance, and grow. Although collaborating with others may be useful, they may also want to find a way to stand out and compete with others so that they make a mark in today’s competitive career landscape. Accordingly, driven by growth, younger workers should also be motivated to strive for status. Older workers, in contrast, may be less motivated by promotions, instead prioritizing stability and maintaining existing achievements instead of competing for higher status (see Carstensen et al., 1999). Furthermore, maintenance goal orientation may involve coaching and collaborating with others to preserve one’s legacy and pass on one’s skills. Striving for status may go against these goals as it may involve competing with others, creating hierarchies, and dominating them. The resulting hostility (Anderson et al., 2015) may conflict with the overarching maintenance goal orientations. Inceoglu et al. (2012) and Kooij et al. (2011) found that age was negatively related to extrinsic social motives at work, including competition, power, and status motives at work. We therefore propose:

#### Hypothesis 6

Age is indirectly related to aggregated levels of the status motive, such that: (a) age is negatively related to the status motive through its negative association with growth goal orientation, and (b) age is negatively related to the status motive through its positive association with maintenance goal orientation.

**Fig. 1:**
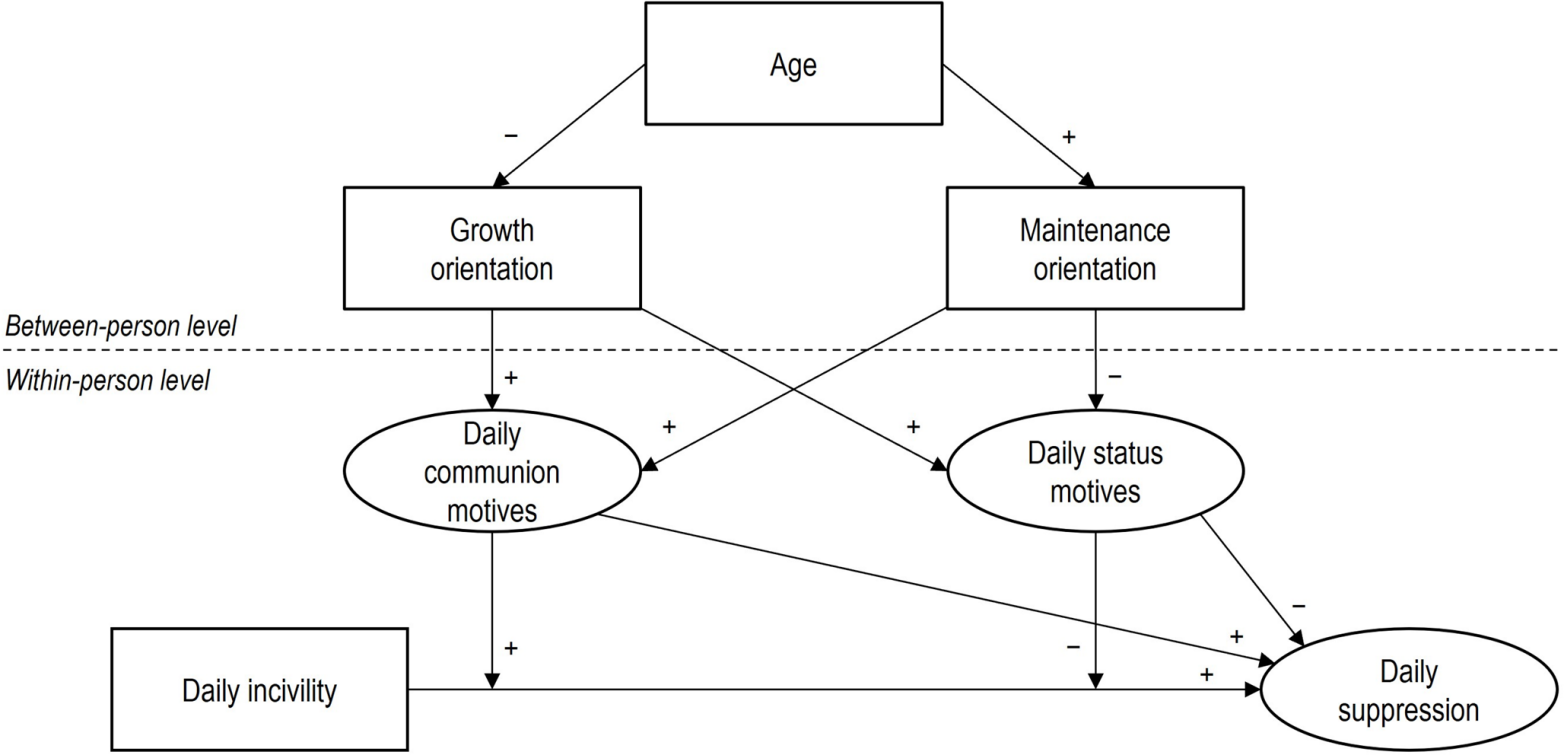
Overarching research model. Latent variables communion motives, status motives, and suppression were allowed to vary at both the within- and between-person levels. We assumed a non-zero correlation between communion and status motives

See Fig. 1 for a summary of our model.

## Method

### Sample and procedure

Dutch participants were recruited via a reputable panel company. The study was part of a larger project on age and work-related emotional competencies and experiences, which consisted of two 30-minute surveys separated by a lag of two weeks (T1 and T2), and a daily diary study approximately two months later consisting of three mini-surveys across 15 working days (T3). Here, we focus on the daily diary study. Eligibility criteria were assessed at T1 and consisted of (1) working 30 h or more per week, (2) working at least 4 days per week, (3) not being involved in shift work, (4) beginning work between 6 and 10 am, and (5) finishing work between 4 and 8 pm. The latter three criteria were set to ensure that people receive invitations for daily diary invites in a way that is synchronous with their workdays.

In the daily diary study, participants had to fill out three surveys per day (lunch, after work, and before bedtime; around 5 min per survey) for 15 working days. We approached 331 participants who provided valid data in T1 and T2 to participate in the daily diary study at T3. Daily diary data were collected in February and March 2021, when some COVID-19 regulations were still in place. We excluded observations when people reported that they did not work on that day or when the daily surveys were filled out outside of the time allotted for that survey (for example, if a person filled out the after-work survey the next morning). This resulted in a total of 3,159 valid daily records. On average, participants participated on 7.87 days (*SD* = 4.32) out of the 15 days. Of all daily observations, 59.7% consisted of data for all three daily entries per day. This sample size and average number of observations per participants is in line with recommendations on daily diary study design by Ohly and colleagues (2010), who report a minimum of 100 participants for person-level predictors and at least five observations per person for day-level predictors.

The mean age of our final sample included in analyses was 44.68 (*SD* = 11.25, 36.1% female). Of all participants, 35% of them were 50 years and older, which is roughly representative of the European workforce (Eurostat, 2024; Kooij et al., 2011). On average, participants worked 8.22 hours per day (*SD* = 0.82), and had been working for their current organization for 11.39 years (*SD =* 9.89). Of the 291 participants, 67.7% had at least a bachelor’s degree or equivalent, and the most common job sectors were business and finance (17.5%), administrative support (11.7%), education and training (9.6%), and management (9.6%). Of all participants, 42.6% reported having a leadership position. Of those, 34.7% reported supervising more than 10 employees.

To compensate them for their efforts, participants received €2 for completing T1, €3.50 for completing T2, and €15, €20, or €30 for completing T3, depending on whether they completed the daily surveys for at least 7, 10, or 13 complete days. The study received ethical approval from the Ethics Committee at the authors’ university and all participants provided informed consent before participating.

### Measures

Table 1 shows descriptive statistics, intraclass correlations (ICCs), reliability coefficient Omegas, and within- and between- variables intercorrelations for all study variables. All items in the daily diary part were adapted to refer to the day-level. All items were administered in Dutch and, when applicable, translated by the authors using the back-translation procedure (Brislin, 1980). Items used for all measures are available on OSF (https://osf.io/zexht/?view_only=c21f7ce2b38048efbbf2331d469cd83d).

**Table 1:** Descriptive statistics and intercorrelations of study variables

	Mean (SD)	ICC	1		2		3		4		5		6		7		
Between-person level																	
1. Age	44.68 (11.25)	−	−														
2. Growth orientation	3.19 (1.00)	−	− 0.19	^***^	−												
3. Maintenance orientation	2.83 (1.13)	−	0.02		0.18	^***^	–										
Within-person level																	
4. Incivility (dichotomous)	0.11 (0.31)	0.30	− 0.05		0.21	^***^	0.30	^***^	-		0.14	^***^	0.06	^**^	0.09	^***^	
5. Suppression	1.74 (1.00)	0.49	− 0.03		0.18	^**^	0.22	^***^	0.47	^***^	(0.87, 0.99)		0.12	^***^	0.05	^*^	
6. Communion motives	2.76 (1.07)	0.55	− 0.02		0.18	^**^	0.01		− 0.14	^*^	0.05		(0.79, 0.90)		0.28	^***^	
7. Status motives	2.20 (1.14)	0.71	− 0.09		0.30	^***^	0.06		0.10		0.18	^**^	0.44	^***^	(0.84, 0.97)		

Note. ICC = intraclass correlation. Correlations below the diagonal are between-person correlations, and correlations above the diagonal are within-person correlations. Values on the diagonal represent the variable’s within-person coefficient omega (before comma), and between-person coefficient omega (after comma)

N (clusters) = 291; N (observations) = 3,159

^*^ *p* ≤ .05

^**^ *p* ≤ .01

^***^ *p* ≤ .001

#### Between-subject measure

Developmental goal orientations were measured at T2 using two scenario descriptions adapted from a career stage measure by Pappas and Flaherty (2006). This measure captures aspects of growth and maintenance goal orientations in the work context.

This scenario measure had been previously used in other studies (Flaherty & Pappas, 2002; Miao et al., 2009) as a practical alternative to relying on temporal variables such as age or tenure to measure career stages. In previous studies, this scale consisted of a categorical measure of career stages in which participants select their stage by selecting one in four passages (exploration, establishment, maintenance, disengagement) based on Cron’s (1984) career stage theory. Here, we measured each item on a five-point Likert scale (1 – completely disagree to 5 – completely agree) to adopt a fluid approach to developmental goal orientations at work (Baruch & Bozionelos, 2011). We measured but did not further consider the exploration and disengagement career stages as we deemed them to be less relevant to our sample of employed adults. Indeed, exploration is theoretically more relevant to young adults who are about to enter the workforce, whereas disengagement may be very specific to some individuals whose retirement is imminent (but see Footnote 2).

#### Within-subject measures

##### Incivility

Incivility was measured in the after-work survey using three items adapted from Hershcovis et al. (2017). For each item, participants indicated the number of times the event happened to them on that day (1 – never to 5 – four times or more). To account for teleworking which was prevalent during the period of data collection due to the COVID-19 pandemic, we adapted the stem to include situations in which incivility occurred both online and offline. A sample item for incivility was ‘This afternoon during work, have you been in a situation in which anyone has (online or offline) behaved rudely to you?’. Participants reported that the incivility was mostly instigated by a colleague (34.4% of daily incivility records), followed by a client (27.6%), a superior (22.2%), and a subordinate (15.4%). Due to the relatively low daily incidence of incivility (10.7% of daily records), we transformed incivility to a dichotomous variable. On days in which incivility occurred at least once, incivility was coded as 1, and on days in which incivility did not occur, incivility was coded as 0. Of all participants in our sample, 112 (40.6%) reported incivility at least once.

### Daily social motives

Communion and status motives were measured in the after-work survey using three items each from Barrick et al.’s (2002) motivational orientation inventory. Participants were asked what they were trying to achieve on that day and rated each item from 1 (not at all) to 5 (to a very high extent). A sample item that measures communion motive is ‘to get along with others at work’, and a sample item for status motive is ‘to get ahead of my co-workers’.

### Expressive suppression

Suppression was measured in the after-work survey using three items adapted from Quigley and Dobson (2014). Participants had to think about how they dealt with their emotions or with the situation when they navigated unpleasant aspects of their job that day by rating each item from 1 (not at all) to 5 (to a very high extent). The items that measured suppression were ‘I tried to suppress my emotions,’ ‘I tried not to let my feelings show,’ and ‘I tried to keep my emotions to myself.’

### Control variables

Suppression was also measured in the lunch survey using one item from Quigley and Dobson (2014). We added this measure as a control variable to control for prior levels of expressive suppression in the analysis. Given the significant negative within-level correlation between day of study and suppression (*r* = − .14, *p* < .001), we also added day of study as a control variable for these analyses.

### Analytical approach

Because daily observations were nested within participants, we adopted a multilevel approach to our analyses using M*plus* 8.0 (Muthén & Muthén, 2017). ICCs of within-level variables ranged from 0.30 to 0.71 (see Table 1). We specified a multilevel factor model in which each variable had one within-level factor and one between-level factor. Items loaded on both the between-level factor and the within-level factor of their corresponding variable. Then, we proceeded with a multilevel confirmatory factor analysis (MCFA) to establish the distinctness of communion motives, status motives, and suppression.

To test our hypotheses, we used multilevel structural equation modelling with Mplus’ Bayesian estimator. We chose the Bayesian estimator as it allowed us to compute interaction terms between observed variables with missing values and latent variables, and because it does not make distributional assumptions about indirect effects (Hox et al., 2014; Preacher et al., 2010). M*plus* default priors were used for the estimation of model parameters. Missing data were dealt with using M*plus*’ Full Information Maximum Likelihood (FIML).

We followed the recommendations of Preacher and colleagues (2010, 2016) to test our model using latent variables. Age was measured at T1 by asking participants to indicate their age. To make the coefficients of age more easily interpretable and comparable to coefficients of other variables, we divided age by 10 (see also Fasbender et al., 2020; Gielnik et al., 2018). We person mean-centered the within-person level variables (day in study, incivility and expressive suppression at lunch time) and grand mean-centered between-person level variables (age, growth goal orientation, and maintenance goal orientation). Communion and status motives were allowed to vary at both levels. In other words, for both communion and status motives, we distinguished between the within-person component and the between-person component. First, we specified a preliminary model without interaction terms (Model 1). At the within-person level, suppression (after work), communion motive (after work) and status motive (after work) were specified as latent factors with three indicators each. We specified three random slopes to regress the within-person level suppression factor on incivility (one indicator), the communion motive factor, and the status motive factor. Additionally, we controlled for day and prior levels of suppression using fixed-effect paths.^1^ At the between-person level, communion and status motives (after work) were specified as latent factors with three indicators each. For completeness, we also specified a between-person level latent factor for suppression. We regressed each of the between-person level motive factors on the two developmental goal orientations (growth and maintenance) and each of the goal orientations on age. We only calculated indirect effects and motives when all paths of the mediation reached significance. In Model 2, we ran a multilevel regression with similar specifications with the addition of two latent interaction terms at the within-person level: one between incivility and the communion motive latent factor, and one between incivility and the status motive latent factor. Using random slopes, we regressed suppression on each of the interaction terms. To create latent interaction terms, we made use of M*plus*’ XWITH function. Note that in all models, we assumed non-zero correlations between status and communion motives (at the within- and between-person levels), and at the between-person level, between suppression and each of communion motive, status motive, age, growth goal orientation, and maintenance goal orientation.

### Data availability

Data and codes used for analyses are available upon request.

## Results

### Descriptive statistics and multilevel CFA

The ICCs ranged from 0.30 (for incivility) to 0.71 (for status). Therefore, we concluded that our constructs show within-person variability across days. A three-factor multilevel CFA resulted in good fit (χ^2^ = 167.74, df = 48, *p* < .001; RMSEA = 0.03; CFI = 0.99; within-level SRMR = 0.02; between-level SRMR = 0.06). We compared this model to a one-factor multilevel CFA in which all items loaded on one within-level factor and one between-level factor, and the fit significantly decreased (χ^2^ = 7170. 802, df = 54, *p* < .001; RMSEA = 0.22; CFI = 0.40; within-level SRMR = 0.22; between-level SRMR = 0.27). This indicates that the constructs are sufficiently distinct from one another on the between- and within-person levels of analysis. Note that at the within-person level, incivility is significantly positively related to both communion (*r* = .06, *p* < .01) and status motives (*r* = .09, *p* < .001).

### Hypothesis testing

Estimates of the analysis are summarized in Tables 2 and 3. At the within-person level (Table 2), incivility predicted suppression use (*B* = 0.30, *p* < .001, one-tailed), thereby providing support for Hypothesis 1. Further, daily communion motive was positively related to suppression (*B* = 0.08, *p* < .01, one-tailed), but daily status motive was unrelated to suppression (*B* = 0.01, *p* = .42, one-tailed). Our results therefore provide support for Hypothesis 2a, but not Hypothesis 2b. Both interactions between incivility and communion and incivility and status were not significant (Model 2); we therefore could not find support for Hypotheses 3a and 3b.

**Table 2:** Unstandardized coefficients of multilevel structural equation model: suppression as outcome variable

	Model 1 (no interaction terms)			Model 2 (with interaction terms)		
	Coefficient	*Posterior SD*	95% CI	Coefficient	*Posterior SD*	95% CI
Day of study	− 0.02^***^	< 0.01	[-0.02, − 0.01]	− 0.02^***^	< 0.01	[-0.02, − 0.01]
Prior levels of suppression	0.16^***^	0.02	[0.12, 0.20]	0.16^***^	0.02	[0.12, 0.20]
**Random slopes**						
Incivility	0.30^***^	0.07	[0.16, 0.45]	0.30^***^	0.07	[0.16, 0.43]
Communion motives	0.08^**^	0.03	[0.02, 0.14]	0.08^**^	0.03	[0.02, 0.14]
Status motives	0.01	0.04	[-0.06, 0.09]	0.02	0.04	[-0.06, 0.10]
Incivility*communion motives	–	-	-	− 0.05	0.16	[-0.33, 0.27]
Incivility*status motives	–	-	-	0.15	0.16	[-0.17, 0.47]
**Within-level residual variance**	0.29^***^	0.02	[0.26, 0.32]	0.30^***^	0.02	[0.26, 0.33]

*Posterior SD * posterior standard deviation. *CI * credibility interval. The Level 2 results of this model are reported in Table 3. Note that Model 2 included 2,610 daily observations.

^**^ *p* < .01. ^***^ *p* < .001 (one-tailed)

At the between-person level (Table 3), age was negatively related to growth goal orientation (*B* = − 0.17, *p* < .01, one-tailed), but unrelated to maintenance goal orientation, thereby providing support for Hypothesis 4a but not 4b. Growth, in turn, was positively related to both communion (*B* = 0.15, *p* < .01, one-tailed), and status motives (*B* = 0.28, *p* < .001, one-tailed), while maintenance was unrelated to communion and status motives^2^. Therefore, we only computed the indirect effects of age on status and communion motives via growth goal orientation. Our results showed that age is indirectly negatively related to communion and status motives via growth, providing support for Hypotheses 5a and 6a. We could not find support for Hypotheses 5b and 6b, given that maintenance was unrelated to either motives.^3^ In this model, the within-person residual variance for suppression was 0.30, compared to a within-person residual variance of 0.39 in the null model. In other words, the model accounted for 9% of intraindividual variability in suppression.

**Table 3:** Unstandardized coefficients of multilevel structural equation model: motives as outcome variables

	Model 1 (no interaction terms)				Model 2 (with interaction terms)		
	Coefficient		Posterior SD	95% CI	Coefficient	Posterior SD	95% CI
**Between-level effects**							
Age → growth	− 0.17^**^		0.05	[-0.27, − 0.07]	− 0.17^***^	0.05	[-0.28, − 0.07]
Age → maintenance	0.02		0.06	[-0.09, 0.14]	0.01	0.06	[-0.11, 0.13]
Growth → communion motives	0.15^**^		0.06	[0.03, 0.26]	0.15^**^	0.06	[0.04, 0.26]
Growth → status motives	0.28^***^		0.06	[0.16, 0.40]	0.28^***^	0.06	[0.16, 0.40]
Maintenance→ communion motives	− 0.01		0.05	[-0.11, 0.08]	− 0.02	0.05	[-0.11, 0.08]
Maintenance → status motives	0.01		0.05	[-0.09, 0.120]	0.02	0.05	[-0.09, 0.12]
Age → communion motives	0.02		0.05	[-0.08, 0.12]	0.01	0.05	[-0.09, 0.11]
Age → status motives	− 0.02		0.05	[-0.13, 0.08]	− 0.02	0.06	[-0.13, 0.08]
**Between-level residual variances**							
Growth	0.99^***^		0.08	[0.85, 1.18]	1.01^***^	0.08	[0.86, 1.20]
Maintenance	1.31^***^		0.11	[1.12, 1.54]	1.32^***^	0.12	[1.12, 1.61]
Communion motives	0.69^***^		0.08	[0.56, 0.86]	0.68^***^	0.07	[0.54, 0.84]
Status motives	0.91^***^		0.09	[0.75, 1.11]	0.91^***^	0.08	[0.75, 1.09]

		Model 1			Model 2		
		Coefficient	Posterior SD	95% CI	Coefficient	Posterior SD	95% CI
**Between-level indirect effects**							
Age → growth → communion motives		− 0.02^**^	0.01	[-0.05, <-0.01]	− 0.03^**^	0.01	[-0.06, − 0.01]
Age → growth → status motives		− 0.05^**^	0.02	[-0.09, − 0.02]	− 0.05^***^	0.02	[-0.09, − 0.02]

*Posterior SD * posterior standard deviation. *CI*credibility interval. Age was measured in decades. The Level 1 results of this model are reported in Table 2. Note that Model 2 included 2,610 daily observations.^**^ *p* ≤ .01. ^***^ *p* < .001 (one-tailed)

## Discussion

In this research, we applied the motivated framework to emotion regulation in the work context to investigate the context around suppression use at work. We looked at (1) what motivates people to use suppression in their daily work (within-person level) and (2) how these motives vary between persons of different ages through developmental goal orientations (between-person level). Our findings show that incivility, a social work stressor, is positively related to suppression use. This is in line with previous studies that found suppression use to be higher in interpersonal situations than when employees are alone (e.g., Diefendorff et al., 2008; Scheibe & Moghimi, 2021; Sliter et al., 2010), and implies that social work stressors may encourage the use of suppression.

In line with our predictions, the daily communion motive was also positively related to suppression. This finding implies that on days when they want to affiliate and collaborate with others, employees may perceive utility for suppression as it may help them achieve these goals. Note that this is in contrast to previous research by Eldesouky and English (2019), who found, across three studies in the general population, no or weak links between suppression and prosocial goals. The different findings suggest that when people want to get along with and be accepted by others at work, they may use different strategies to achieve those motives than when people want to get along with others outside work. In private relationships, people may value closeness and authenticity more, and may therefore be less likely to use suppression, and more likely to use other strategies. Our present findings, when compared with previous findings on suppression outside the work context, therefore contribute to the literature by highlighting the need to better contextualize the use of suppression and by identifying differences between work and personal situations.

We did not find support for our prediction that the daily status motive would be negatively related to suppression. Rather, our findings indicate that the daily status motive was unrelated to suppression, which may be due to the way we measured the status motive. In fact, Cheng et al. (2013) argue that people strive for status through two different means: prestige, which is conceptualized as gaining respect and status through demonstrating and sharing one’s knowledge and skills, and dominance, which is conceptualized as gaining respect through intimidating others and forcing them to subordinate. Our measure of status captures the former facet, which implies that people who score high on this measure are also achievement oriented. Therefore, although status may encourage people to regulate their affect through the use of more expressive strategies, they may be less likely to spend resources regulating their emotions, as affective regulation may interfere with their work-related performance goals (Beal et al., 2005).

We did not identify any interaction effects between incivility and communion or status motives on suppression. One potential explanation for these non-significant results is that the psychological processes that lead to suppression from incivility and the communion motive are independent from one another. For example, it could be that incivility leads to suppression via lowered self-esteem and sociometric standing, whereas being motivated by communion may lead to suppression via wanting to collaborate smoothly with colleagues. Another explanation could be that suppression in response to incivility is reactive, whereas suppression as a result of motives could be more pro-active and deliberate, which makes the two mechanisms independent of each other.

At the between-person level, and consistent with our assumptions, older workers were less likely than younger workers to be growth oriented. This is in line with theoretical work that predicts that age should be negatively associated with growth (Baltes et al., 2007). Further, growth was positively related to both status and communion motives and age was negatively related to communion and status motives via growth. These results are consistent with the meta-analytic findings of Kooij et al. (2011), who found that older workers were less likely to be motivated by affiliation (i.e., working with others). These findings are also in line with aging theories which postulate that older workers are likely to want to prioritize relationships with close and familiar others such as friends, and not towards more peripheral relationships like coworkers, as the value of these relationships may be lower for older workers (Antonucci & Akiyama, 1987; Carstensen et al., 1999).

Contrary to our expectation, age was unrelated to a maintenance goal orientation. This is in contrast with lifespan and career development research(e.g., Baltes et al., 2007; Cron, 1984) which predicts that older workers should more likely be in career stages that are geared towards maintenance and loss regulation. One potential explanation for this finding could be that career trajectories become more heterogeneous over time, as some people follow straight career trajectories while others change career paths mid-career or get involved in non-work roles like parenting. This is in line with previous research that suggests that older workers are more heterogeneous than younger workers (Scheibe & Kooij, 2024). Moreover, maintenance did not explain any variability in daily social motives. This could be because people who are maintenance oriented are less dependent on others at work compared to those who are growth oriented, presumably because people with maintenance orientation already established themselves and built up the needed resources. In fact, as our study shows, people who are growth oriented are more likely to be young and at earlier stages in their careers. Therefore, they are in the process of building work-related resources, including the accumulation of social capital (Seibert et al., 2001), in order to pave the way for career success.

### Theoretical implications

Our findings offer several theoretical insights. First, by looking at how motives are related to suppression use in the work context, our study illustrates the value of adopting a contextual approach to emotion regulation strategies (see also Isaacowitz & English, 2024). Even though the idea that emotion regulation is motivated is not new (Eldesouky & English, 2019; Tamir, 2016), little research has paid attention to why people regulate their emotions in the work context, although people pursue a number of instrumental, non-hedonic goals at work. Here, we show that emotion regulation strategies, such as suppression, may be modulated by social work motives, and therefore related to the self-regulation of instrumental goals. This perspective on emotion regulation at work may therefore complement prevalent and traditional perspectives of workplace emotion regulation which understand emotion regulation as a process that regulates work-related stress in order to maintain well-being (Gabriel et al., 2015; Scheibe et al., 2016; Scheibe & Moghimi, 2021; Shimazu & Kosugi, 2003).

Second, our results highlight the importance of testing differences in how age-related processes manifest across different contexts or life domains (e.g., personal life vs. work). In fact, our results show that relationships between age and social motives may be explained by a lower inclination to be growth oriented, which can be understood as a unique manifestation of age-related motivational shifts in the work context. Further, although age is thought to be associated with higher prioritization of social and affiliative goals (Carstensen et al., 1999), our results show that this may not apply in all contexts, such as the work context. Therefore, future research should test how postulations derived from lifespan theories may play out in multiple life contexts, including the work context (Baltes et al., 2007).

Third, our results show that in the work context, older workers may be less likely to reallocate their resources for growth, but not necessarily more likely to reallocate their resources for maintenance. This could be because samples with older workers tend to be younger than samples with older adults. Note that our sample comprised a significant number of older workers which is representative of the proportion of older workers (50+) in the European workforce (Eurostat, 2024). Consequently, allocating resources for the purposes of maintenance or loss regulation may occur later in the working lifespan, and perhaps only for a subgroup of older workers who suffer from health problems and are at risk to retire prematurely (see Sundstrup et al., 2024). Moreover, older workers may represent a particularly healthy proportion of the population that was able to stay in the workforce for longer (McMichael, 1976), and this may explain why we do not find the expected pattern of increased maintenance and loss regulation orientation for older adults. Overall then, research linking broader developmental growth orientations to daily motives related to emotion regulation strategies at work helps address calls to improve our understanding of *why* (rather than how) younger and older workers regulate their emotions in different life contexts (for a review, see Isaacowitz & English, 2024).

### Limitations and future directions

First, although we used a rigorous daily diary design spanning three weeks with three measurement points per day, we could not investigate any chronic or longitudinal effects of incivility or motives on suppression. For instance, even though people were more likely to suppress their emotions on days when they experienced incivility, the way people would regulate their emotions to chronic incivility may be different. For example, Cortina and Magley (2009) found that people who experienced incivility over a longer period of time were more likely to speak out by seeking social or organizational support or by confronting the instigator. Further, although daily communion motives may be positively related to daily suppression, these results may play out differently over a longer span of time. On the long-term, people with communion motives may develop closer relations with their coworkers, which may render them more likely to trust coworkers and rely on them for support (Ferris et al., 2009). As such, they may be less likely to suppress their emotions when facing stressors.

Second, although we found that people may use suppression when they want to get along with others at work, we did not test whether suppression as an emotion regulation strategy helped achieve these goals as the focus of this paper was to examine antecedents of suppression. English et al. (2012), for instance, found that people who habitually used suppression were less liked by college peers four years later. Accordingly, future research should assess how and when motivated regulation strategies relate to the achievement and fulfilment of a person’s goals and motives at work by examining relevant outcome variables.

A third limitation is that we collected the data during the COVID-19 pandemic. Although waves T1 and T2 of the study (in which age and growth/maintenance orientations were recorded, respectively) were collected during relatively strict lockdown measures (e.g., schools and restaurants are closed), T3 (daily diary phase) was collected at a later stage when most measures were relaxed. During the daily diary data collection phase, certain establishments (e.g., restaurants) were still closed, but children were allowed to go to school and employees to their workplaces. However, most observations in the daily diary study (64.4%) were records of people working from home, which may influence the way people experience stressors (e.g., incivility), and respond to them. Incivility, suppression, communion, and status motives are probably more pronounced in location work, as all four variables are related to the relationship between the person and their social work environment. As such, we believe that our design may have been a more conservative test of our hypotheses. However, future research should investigate and extend our research questions in a context in which (most) people are working on location.

Fourth, we focused on suppression as a single emotion regulation strategy, and did not take into account the idea that people may use multiple regulation strategies at a time (Aldao & Nolen-Hoeksema, 2013; Diefendorff et al., 2019; Gabriel et al., 2020). Therefore, daily motives may elicit unique combinations of emotion regulation strategies, including suppression combined with other strategies like reappraisal, or more confrontative forms of coping, such as assertion and seeking formal/informal support (Cortina & Magley, 2009). Therefore, future research should consider the effects of motives and stressors on constellations of emotion regulation strategies by adopting, for example, a person-centered approach.

Fifth, we assumed in this paper a homogeneous view of career development in the workplace, assuming (and finding) that older workers tend to be less motivated by growth. However, interindividual heterogeneity is an important factor to consider in aging workers. Indeed, as workers age, they may acquire more (or less) resources over time (Hobfoll et al., 2018). For example, some may achieve managerial positions while others may be less successful at doing that, or may choose not to climb up the career ladder. This heterogeneity may have some impact on work-related goal orientations. Accordingly, it is important that future research looks at the effect of age along with other relevant variables (e.g., managerial status, organizational tenure, political skill, work-related skills…) on goal orientations (see also Scheibe & Kooij, 2024).

Sixth, it is important that future research investigates the temporal order of the relationships between incivility, motives, and use of suppression at work. This can be achieved through the use of longitudinal studies, even though we caution against long time-lags between situations, motives and emotion regulation strategies as this could lead to false negatives, or even spurious effects. In fact, drawing on major theories of stress and emotion regulation (Gross, 2015; Lazarus & Folkman, 1987), situational appraisals (in terms of situation x motives) and emotion regulation efforts occur simultaneously as the stress/emotion generation process is unfolding, and do not occur after a longer period of time (see also Dormann & Griffin, 2015 for a discussion on optimal time lags in panel studies). Alternatively, causal effects can be achieved using experimental studies. Following the suggestions of a reviewer, we conducted an experimental vignette study to test the relationship between social work situations, motives, and suppression use. Results can be found in the supplementary materials.

## Conclusion

We adopted a motivated perspective to emotion regulation to investigate why people use suppression at work, and whether there are age-graded tendencies to experience work-related motives that relate to suppression. Our findings show that people are more likely to use suppression to regulate their emotions on workdays when they experience incivility and on days when their communion motives are salient. We also find that communion motives are most salient for people who scored higher on growth orientation, which tended to be younger workers. All in all, our results highlight the utility of adopting a motivated view to emotion regulation in the work context to understand why people use a potentially costly emotion regulation strategy at work. Further, our results show the importance of understanding how these social motives vary at the dispositional level across different employee groups.

## Data Availability

Data and codes used for analyses are currently available upon request.

## Footnotes

1 Note that we also tested a model in which we controlled for gender at the between-person level, and daily work location at the between- and within-person levels (to account for hybrid working and teleworking). However, neither of these variables were significantly related to suppression, so we dropped them.

2 Because we also collected data pertaining to two other goal orientations, namely exploration and disengagement, we also tested the model with the addition of these two constructs. Exploration was not significantly related to communion motives (*B* = − 0.02, *posterior SD* = 0.05, one-tailed *p* = .34), nor status motives (*B* = − 0.05, *posterior SD* = 0.06, one-tailed *p* = .20). Similarly, disengagement was also unrelated to communion motives (*B* = < 0.001, *posterior SD* = 0.05, one-tailed *p* = .50) and status motives (*B* = − 0.02, *posterior SD* = 0.06, one-tailed *p* = .36). Age was negatively related to exploration (*B* = − 0.14, *posterior SD* = 0.06, one-tailed *p* = .01) but not disengagement (*B* = 0.02, *posterior SD* = 0.06, one-tailed *p* = .34).

3 We also ran an extended model in which we also regressed suppression on communion and status at the between-person level and in which we added interaction effects between within-person level suppression and between-person level motives (both communion and status) on suppression. Our results showed that neither communion nor status were related to suppression at the between-person level. Both cross-level interactions were non-significant. The significant main effect of within-person communion on suppression remained significant.
